# Focal segmental glomerulosclerosis in which urinary protein improved after surgical treatment for acromegaly: a case report

**DOI:** 10.1186/s13256-019-2228-z

**Published:** 2019-09-21

**Authors:** Arina Yamasaki, Daisuke Bito, Erina Eto, Keiichiro Matsumoto, Megumi Nakamura, Junji Miyazaki, Kenichi Matsumoto, Masanori Masuda, Daisuke Mori, Toru Yoshimura

**Affiliations:** 1 Diabetes and Endocrinology, Saga-Ken Medical Centre Koseikan, 400 Nakabaru, Kase-machi, Saga-shi, Saga, 840-8571 Japan; 2 Nephrology, Saga-Ken Medical Centre Koseikan, Saga, Japan; 3 Otorhinolaryngology, Saga-Ken Medical Centre Koseikan, Saga, Japan; 4 Neurosurgery, Saga-Ken Medical Centre Koseikan, Saga, Japan; 5 Diagnostic Pathology, Saga-Ken Medical Centre Koseikan, Saga, Japan

**Keywords:** Acromegaly, Focal segmental glomerulosclerosis, Growth hormone, Insulin-like growth factor 1

## Abstract

**Background:**

Focal segmental glomerulosclerosis is characterized by partial (segmental) sclerotic lesions in some glomeruli (focal). Primary focal segmental glomerulosclerosis is generally considered resistant to steroid therapy. However, acromegaly is a disease that causes peculiar facial features, body types, and metabolic abnormalities due to the excessive secretion of growth hormone by a pituitary adenoma. Growth hormone has been reported to be involved in glomerular cell growth, mesangial proliferation, and glomerulosclerosis in the kidney.

**Case presentation:**

We report a case of a Japanese patient with focal segmental glomerulosclerosis in whom decreased urinary protein was observed after surgical treatment for acromegaly.

**Conclusion:**

The patient’s urinary protein improved as the concentration of growth hormone/insulin-like growth factor 1 decreased.

## Introduction

Acromegaly is a disease that causes peculiar facial features, body types, and metabolic abnormalities due to the excessive secretion of growth hormone (GH) and insulin-like growth factor 1 (IGF-1). Acromegaly is often complicated by diabetes mellitus, hypertension, and heart disease, and the prognosis is poor in patients who are untreated. However, improvements in diabetes mellitus, hypertension, and heart disease after treatment for acromegaly have been reported [1]. Control of GH hypersecretion, hypertension, and heart disease is therefore needed in order to improve the ultimate mortality rates [2, 3].

GH affects the organs of the whole body, and in the kidney, it is associated with glomerular cell growth, mesangial proliferation, and glomerulosclerosis. Acromegaly is characterized by glomerular hyperfiltration, and the urinary albumin level is high, suggesting the influence of GH and IGF-1 levels [4].

In contrast, focal segmental glomerulosclerosis (FSGS) is characterized by partial (segmental) sclerotic lesions in some glomeruli (focal). Primary FSGS is generally considered to be resistant to steroid therapy. Cases of acromegaly complicated by FSGS are rare. We report a case of a patient with FSGS in whom the urinary protein level improved as the concentration of GH/IGF-1 decreased after surgical treatment for acromegaly.

## Case presentation

The patient was a 64-year-old Japanese man who was admitted to our hospital for the gradual growth of a thyroid tumor. At 20 years of age, urinary protein had been detected in his urine specimen. He became conscious of lip enlargement and a limb volume increase at 30 years of age. He had been diagnosed with diabetes mellitus at 56 years of age, and increased urinary protein was detected at the same time. A renal biopsy was performed. The histopathological findings showed FSGS. However, steroid treatment was not started, owing to diabetes mellitus. A thyroid tumor was detected at 59 years of age, and fine-needle aspiration cytology was performed. No malignant cells were detected in a histopathological examination; however, the thyroid tumors gradually grew larger, and thereafter the patient was introduced to our department. He was suspected of having acromegaly due to his characteristic facial features, and he was therefore admitted to our hospital.

He had previously undergone surgery for the treatment of colon cancer at 61 years of age.

His mother and grandfather had a history of hypertension and diabetes mellitus, respectively. There was no history of endocrinological disease in his family.

His physical examination on admission showed that his height and body weight were 164.0 cm and 65.8 kg, respectively, and he had a body mass index of 24.4 of kg/m^2^. He received losartan potassium 12.5 mg and amlodipine 5 mg as treatment for hypertension. His blood pressure was 108/54 mmHg. His heart rate was 88 beats/minute with sinus rhythm. He showed acromegalic features, including an outstanding jaw and eyebrow area and enlargement of the nose, tongue, and lip. He also had large hands and feet. His thyroid gland was not enlarged, and the thyroid tumor could not be palpated. Pitting edema was not observed in the lower limbs.

**Fig. 1:**
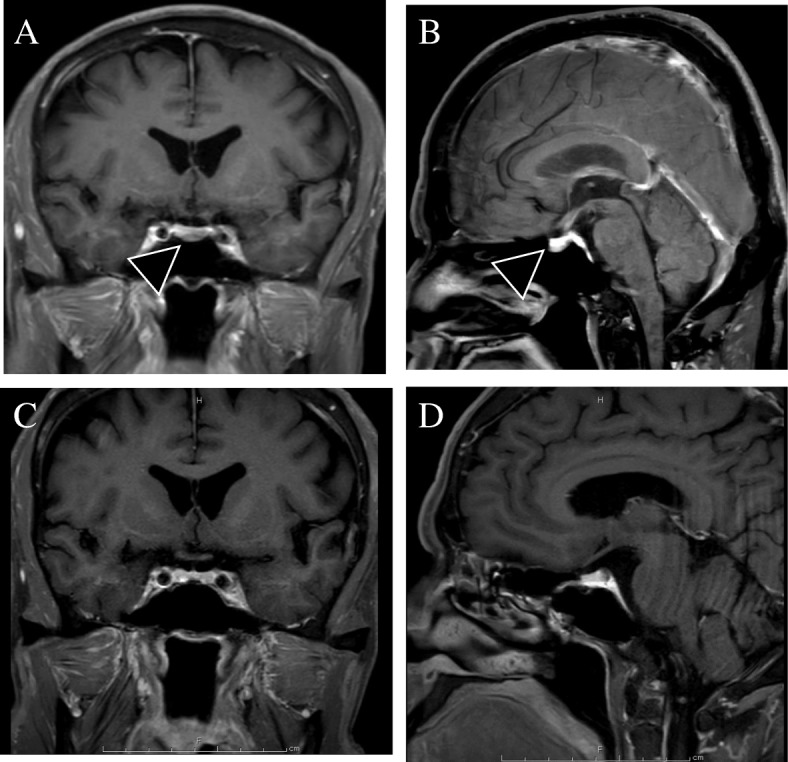
Gadolinium-enhanced pituitary magnetic resonance imaging. **a** Coronal section. **b** Sagittal section (before surgery). **c** Coronal section. **d** Sagittal section (after surgery). Arrow showed a pituitary adenoma 11 mm in diameter

**Fig. 2:**
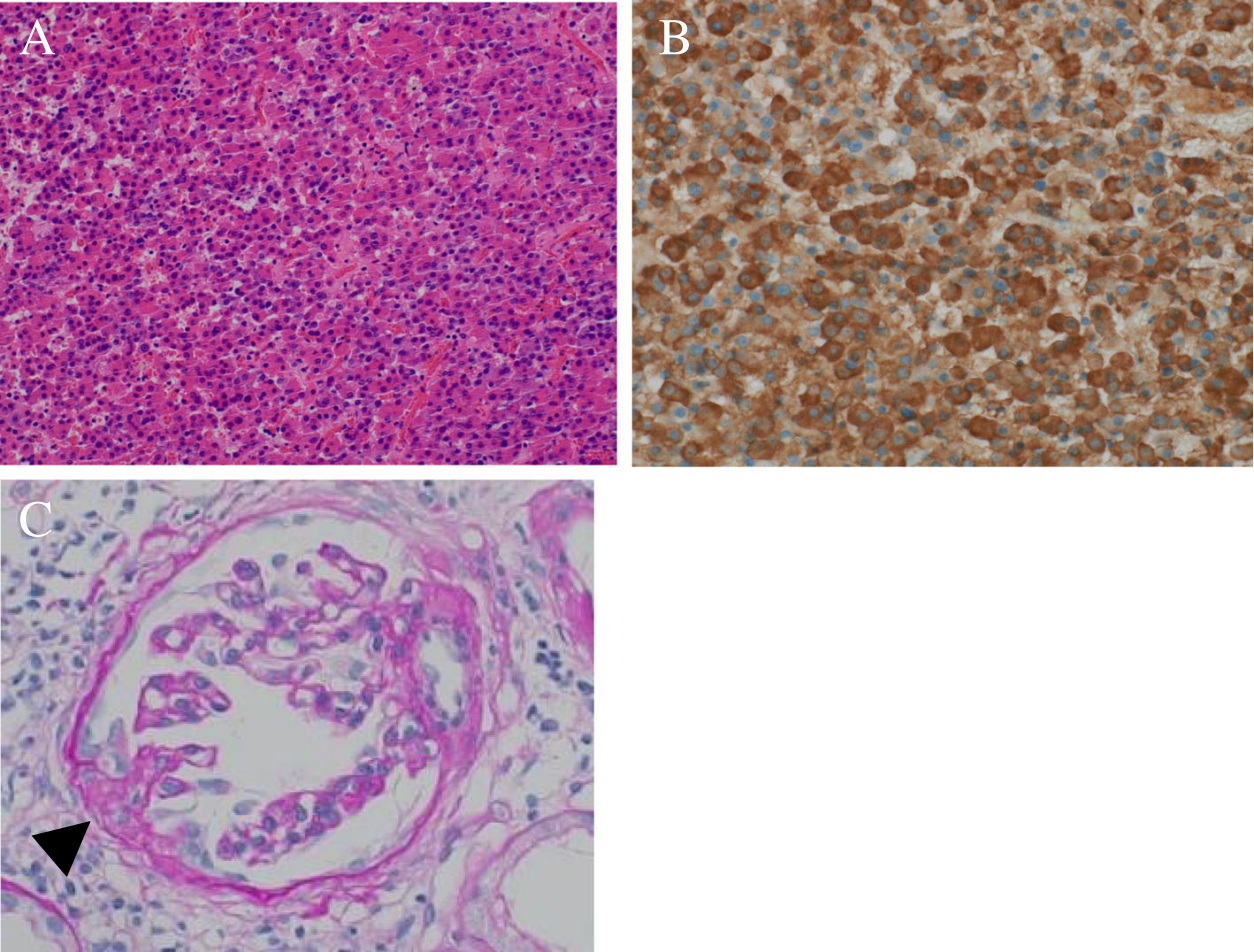
**a** Hematoxylin and Eosin (H&E) staining (original magnification, ×40) shows pituitary adenoma. **b** Growth hormone (GH) staining (original magnification, ×400) shows GH-producing pituitary adenoma. **c** A renal biopsy specimen showing tip variant (*arrow*) (periodic acid-Schiff staining; original magnification, ×400)

Table 1 shows the laboratory findings on admission. Urinalysis showed 2+ protein due to FSGS. The patient’s hemoglobin A1c (HbA1c) level was 6.3% on diet therapy alone. The patient’s lipid levels were within normal limits under treatment with atorvastatin 10 mg. His blood cell counts and blood chemistry were within normal limits. The patient’s GH and IGF-1 levels were 2.7 ng/ml and 496 ng/ml, respectively. His thyroid-stimulating hormone level was 0.03 μIU/ml, but his free T4 level was in the normal range, and his thyroglobulin antibody and thyrotropin receptor antibody were negative. Other pituitary hormones, including luteinizing hormone, follicle-stimulating hormone, and adrenocorticotropic hormone, were all within normal limits. GH was not suppressed by a 75-g oral glucose tolerance test (Table 2), and it was increased paradoxically in response to an intravenous injection of thyrotropin-releasing hormone. Gadolinium-enhanced pituitary magnetic resonance imaging (T1-weighted) showed a pituitary adenoma of 11 mm in diameter (Fig. 1a, b). These findings were consistent with acromegaly. Transsphenoidal surgery was performed to resect the pituitary adenoma. The pituitary adenoma was completely excised (Fig. 1c, d). The histopathological findings showed pituitary adenoma (Fig. 2a). The sections were analyzed with GH staining and diagnosed as GH-producing pituitary adenoma (Fig. 2b). After surgery, the patient’s GH and IGF-1 levels normalized to 0.37 ng/ml and 171 ng/ml, respectively. GH was found to be suppressed on the basis of a 75-g oral glucose tolerance test (Table 2). The treatment was considered to have successfully led to the remission of acromegaly.

**Table 1:** Laboratory findings on admission

Blood cell counts		Blood chemistry		Endocrinology		Urinalysis
WBC	4800/μl	TP	6.2 g/dl	ACTH (7.2–63.3)	18.7 pg/ml	Glucose (−)
Ne	57.5%	Alb	3.4 g/dl	Cortisol (3.7–19.4)	5.9 μg/dl	Protein (2+)
Ly	33.9%	BUN	13.2 mg/dl	TSH (0.4–4.5)	0.03 μIU/ml	Ketone (−)
Mo	5.9%	Cr	0.6 mg/dl	Free T4 (0.9–1.5)	1.26 ng/ml	
Eosinophils	2.5%	eGFR	103 ml/min/1.73 m^2^	TgAb	(−)	
Ba	0.2%	UA	4.3 mg/dl	TRAb	(−)	
RBC	398 × 10^4^ /μl	T-Bil	0.7 mg/dl	PRL (4.3–13.7)	8.31 ng/ml	
Hb	12.6 g/dl	AST	16 IU/L	LH (1.7–8.6)	3.69 mIU/ml	
Hct	38.8%	ALT	12 IU/L	FSH (1.5–12.4)	13.68 mIU/ml	
MCV	97.5 fl	LDH	188 IU/L	Testosterone (1.3–8.7)	5.61 ng/ml	
MCH	31.7 pg	ALP	259 IU/L	GH (< 2.47)	2.7 ng/ml	
MCHC	32.5%	γ-GTP	24 IU/L	IGF-1 (73–224)	496 ng/ml	
PLT	22.5 × 10^4^/μl	ChE	369 IU/L	AVP (< 2.8)	1.0 pg/ml	
		AMY	145 IU/L			
		LDL-C	79 mg/dl			
		HDL-C	79 mg/dl			
		TG	81 mg/dl			
		Na^+^	142 mEq/L			
		K^+^	4.2 mEq/L			
		Cl	109 mEq/L			
		Ca^2+^	9.1 mg/dl			
		P	3.5 ng/ml			
		Glu	104 mg/dl			
		HbA1c	6.3%			

*ACTH* Adrenocorticotropic hormone, *Alb* Albumin, *ALP* Alkaline phosphatase, *ALT* Alanine aminotransferase, *AST* Aspartate aminotransferase, *AVP* Arginine vasopressin, *BUN* Blood urea nitrogen, *Ca*^*2+*^ Calcium, *ChE* Cholinesterase, *Cl* Chloride, *Cr* Creatinine, *eGFR* Estimated glomerular filtration rate, *FSH* Follicle-stimulating hormone, *GH* Growth hormone, *Glu* Glucose, *γ-GTP* γ-Glutamyl transpeptidase, *Hb* Hemoglobin, *HbA1c* Hemoglobin A1c, *Hct* Hematocrit, *HDL-C* High-density lipoprotein cholesterol, *IGF-1* Insulin-like growth factor 1, *K*^*+*^ Potassium, *LDH* Lactate dehydrogenase, *LDL-C* Low-density lipoprotein cholesterol, *LH* Luteinizing hormone, *MCH* Mean corpuscular hemoglobin, *MCHC* Mean corpuscular hemoglobin concentration, *MCV* Mean corpuscular volume, *Na*^*+*^ Sodium, *PLT* Platelets, *PRL* Prolactin, *RBC* Red blood cells, *T4* Thyroxine, *T-Bil* Total bilirubin, *TG* Triglycerides, *TgAb* Thyroglobulin antibody, *TP* Total protein, *TRAb* Thyrotropin receptor antibody, *TSH* Thyroid-stimulating hormone, *UA* Urinalysis, *WBC* White blood cells, *AMY* Amylase, *Ba* Basophil, *Ly* Lymphocyte, *Mo* Monocyte, *Ne* Neutrophil, *P* Phosphorus

**Table 2:** The 75-g oral glucose tolerance test results before and after surgery

	0 minutes	30 minutes	60 minutes	120 minutes
Before surgery				
GH (ng/ml)	3.39	3.87	7.39	7.64
After surgery				
GH (ng/ml)	0.38	0.35	1.37	0.32

*GH* Growth hormone

Figure 2c shows a renal biopsy performed at 58 years of age. A glomerulosclerotic lesion was found at the proximal tubule on the contralateral side of the glomerular vascular pole (tip variant). At this time, the patient was diagnosed with FSGS.

During hospitalization after the surgery, his cardiovascular examination findings were normal; his lungs were clear to auscultation; and the result of his abdominal examination was unremarkable. The peculiar facial features found in acromegaly did not change. The result of his neurological examination was completely unremarkable. No abnormal findings appeared after the surgery.

**Fig. 3:**
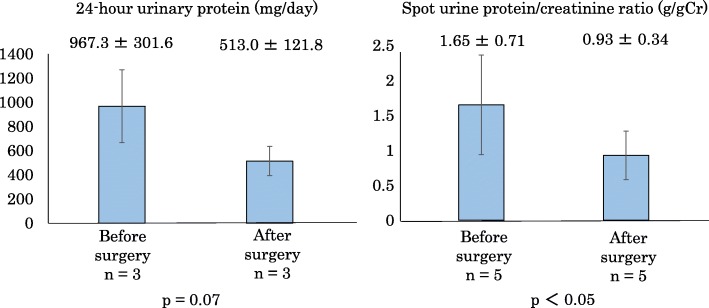
Changes in urinary protein

Urinalyses were performed before and after the surgery; the data are shown in Fig. 3. Twenty-four-hour urine collection was performed three times before and three times after the surgery. The patient’s urinary protein decreased from 967.3 ± 301.6 mg/day before the surgery to 513.0 ± 121.8 mg/day at 3 months after the surgery (*p* = 0.07). We followed the patient every 2 months for 8 months after the surgery in order to measure the spot urine protein/creatinine (Cr) ratio and HbA1c level. The patient’s spot urine protein/Cr ratio significantly improved from 1.65 ± 0.71 g/g Cr before the surgery to 0.93 ± 0.34 g/g Cr after the surgery (*n* = 5 at both points; *p* < 0.05). The patient’s HbA1c level significantly improved from 6.32% ± 0.1% before the surgery to 6.08% ± 0.1% after the surgery (*n* = 5 at both points; *p* < 0.05).

## Discussion

FSGS is characterized by partial (segmental) sclerotic lesions in some glomeruli (focal). FSGS accounts for approximately 10.0% of all cases of adult primary nephrotic syndrome in Japan. Currently, there is no well-established treatment for primary FSGS. Primary FSGS is generally considered resistant to steroid therapy [5]. When a patient’s condition is resistant to steroid treatment, immunosuppressants are used in combination with steroids. Antihypertensive agents, antiplatelet agents/anticoagulants, and lipid abnormality-ameliorating agents are used as adjuvant therapy. Angiotensin-converting enzyme inhibitors (ACEIs) and angiotensin II receptor blockers (ARBs) are expected not only to lower blood pressure but also to reduce urinary protein. Thus, ACEI and ARB antihypertensive agents are administered as first-line treatment [6, 7].

Various mechanisms are involved in the development and progression of glomerular lesions, and recently attention has been paid to the relationship between IGF-1 and glomerular hypertrophy. IGF-1 is produced mainly in the liver, but some IGF-1 is produced in kidney mesangial cells and renal tubules [8, 9]. The GH/IGF-1 axis promotes renal hypertrophy, and the glomerular filtration rate (GFR) accelerates. Mild glomerular hypertrophy is observed in mice transgenic for IGF-1. Mice transgenic for GH show high glomerular hypertrophy and glomerulosclerosis. GH is involved in glomerular cell enlargement and glomerular sclerosis in the kidney [10]. Most studies on the relationship between IGF-1 and renal disease are related to diabetes mellitus or hyperglycemia. Glomerular hypertrophy is observed in early diabetic nephropathy, and the GH/IGF-1 axis plays a major role in its development. IGF-1 is transiently increased in the kidney before renal hypertrophy in early diabetic nephropathy. Diabetic rats with reduced GH secretion showed less renal and glomerular hypertrophy than a control group [11]. Wild-type mice showed pathological changes in diabetic nephropathy, such as glomerular volume increase, an increased mesangial area, and glomerulosclerosis, whereas mice deficient in GH receptor gene showed protection against streptozotocin diabetes-induced nephropathy [12]. In studies in which somatostatin analogs were administered to diabetic animal models, IGF-1 decreased, glomerular hypertrophy was suppressed [13–15], and microalbuminuria decreased [16, 17]. In humans, diabetes mellitus is associated with the GH/IGF-1 axis, GFR, renal hypertrophy, and microalbuminuria [18, 19]. The administration of somatostatin analogs to patients with type 1 diabetes mellitus resulted in improvement of the GFR and kidney size [20]. The administration of somatostatin analogs to patients with type 1 diabetes mellitus has also been reported to reduce serum IGF-1 and urinary albumin [21].

Acromegaly is a disease that causes peculiar facial features, body types, and metabolic abnormalities as a result of the excessive secretion of GH and IGF-1. Acromegaly is characterized by remarkable structural features and functional changes in the kidney that occur because of excess GH; these include renal hypertrophy and renal hyperfiltration [22]. The reduction of GH after the surgical treatment of acromegaly improves the GFR and reduces the size of the kidney [23, 24]. In one case report, a recurrence of nephrotic syndrome was suppressed after the injection of octreotide acetate and the performance of transsphenoidal surgery in a patient with acromegaly complicated by steroid-treated FSGS [25].

In our patient, steroids were not administered, owing to diabetes mellitus, and he was treated with only an ARB. We did not change the antihypertensive agents before or after surgery, and we have not started administering oral antidiabetes drugs or steroid therapy. Thus, we considered that the patient’s urinary protein improved as the concentration of GH/IGF-1 decreased.

## Conclusion

We report a case of a patient with FSGS in whom decrease of urinary protein was observed after surgical treatment for acromegaly. The patient’s urinary protein improved as the concentration of GH/IGF-1 decreased.

## Data Availability

Not applicable.
